# Procedure Prioritization During a Nationwide Ban on Non-Urgent Healthcare: A Quasi-Experimental Retrospective Study of Hospital Data in Switzerland

**DOI:** 10.1177/11786329241293534

**Published:** 2024-10-23

**Authors:** Thomas Grischott, Tarun Mehra, Matthias R Meyer, Oliver Senn, Yael Rachamin

**Affiliations:** 1 Institute of Primary Care, University Hospital Zurich, University of Zurich, Zurich, Switzerland; 2 Department of Medical Oncology and Hematology, University Hospital Zurich, Zurich, Switzerland; 3 Division of Cardiology, Cantonal Hospital Graubünden, Chur, Switzerland

**Keywords:** Treatment prioritization, capacity planning, healthcare access, health equity, COVID-19, Switzerland

## Abstract

**Background:**

During the COVID-19 lockdown in spring 2020, Switzerland restricted non-urgent healthcare services to safeguard capacity. While prioritization of care was supposed to be driven by medical urgency, demographic factors or economic incentives might have influenced the hospitals’ resource allocation decisions.

**Objectives:**

This study investigates potential determinants of procedure prioritization in hospitalized patients during the lockdown period.

**Design:**

Quasi-experimental retrospective study of hospital data in Switzerland.

**Methods:**

We analyzed 496 456 adult patients with known insurance status and a recorded procedure, admitted for cardiovascular, orthopedic/musculoskeletal or oncological reasons from January 2017 (3 years before the COVID-19 outbreak) to mid-April 2020 (in the first year of the COVID-19 pandemic), to obtain admission rate ratios (ARRs, “lockdown” admission rates divided by “normal” rates) from negative binomial regression analysis of fortnightly admissions for frequent procedure-diagnosis combinations. Quade and Wilcoxon signed-rank tests compared ARRs between sex×age, insurance and comorbidity strata.

**Results:**

Admission rates showed significant reductions for 29 of 53 procedure-diagnosis combinations. Reductions varied strongly by emergency, with largest decreases in orthopedic procedures for arthrosis (osteoarthritis) and non-arthritic joint disorders, and the smallest in cerebral imaging for stroke patients and surgical procedures for malignant neoplasms. The only difference in ARRs between strata was a stronger decrease in admission rates for cardiovascular combinations for patients with private versus basic health insurance.

**Conclusion:**

While medical procedures were affected to varying degrees by the ban on non-urgent healthcare during the COVID-19 lockdown, we found no robust evidence that factors other than medical urgency influenced healthcare prioritization.

## Introduction

The principle of equal access to healthcare based on need has been adopted as a major policy objective by most OECD countries but its actual implementation remains challenging.^1^ Disparities in healthcare access may arise from financial barriers or differences in patients’ health literacy, but also from differential availability of healthcare services to certain patient groups, for example when providers are unequally inclined to offer treatment to patients with identical needs but from different populations.^1^

The COVID-19 pandemic represents a natural experiment that allows to test the viability of the principle of horizontal equity (ie, equal access for equal need) under constrained availability of resources and increased pressure on the healthcare system. In the spring of 2020, the provision of healthcare services had to be restructured in order to free up capacities for COVID-19 patients. In Switzerland, this entailed restricting non-urgent healthcare services from 16 March 2020 to 27 April 2020. During this government-imposed ban, the “healthcare resource” thus became scarce(r), whilst the healthcare needs of the majority of the population remained, at best, constant. Consequently, “non-urgent” procedures were cancelled or postponed,^2^ but the criteria for prioritization and what was considered “urgent” were only vaguely defined. The Central Ethics Committee of the Swiss Academy of Medical Sciences (SAMW) published a rather general list of criteria considered “unfair” and therefore inappropriate.^3^ In general, urgency or medical need are not usually characteristics of a specific procedure, but of a specific treatment in a specific situation, which is largely co-determined by the diagnosis and often other variables such as, for example, the severity of the disease or the presence of comorbidities.

Moreover, urgency may not have been the only factor in prioritizing healthcare services. In particular, economic considerations might have posed a threat to purely needs-based prioritization. When capacity is scarce, economic incentives could work in favor of certain patient groups and to the disadvantage of others whose treatment is less lucrative.^4^ Indeed, Swiss hospitals are mainly financed through a diagnosis-related groups (DRG)-based reimbursement system, which penalizes lengthy hospital stays and may therefore tend to disfavor hospitals caring for highly complex and comorbid patients. Moreover, hospitals tend to profit more from privately and semi-privately insured patients than from patients with general insurance.^5,6^ Although the same flat rates are charged for all patients under compulsory health insurance in Switzerland, regardless of their insurance status, private health insurers pay substantial surcharges for treatment by the head physician, for single room accommodation or for higher quality, more expensive meals. Previous work has shown that patients with private health insurances were more likely to receive total hip arthroplasty in the US and Switzerland,^7,8^ and that the insurance status was also associated with arthroscopic knee surgery in Switzerland.^9^ It seems possible that such effects may have exacerbated in times of scarcer resources.

With this study, we thus aimed to explore (determinants of) prioritization of inpatient procedures during the COVID-19 ban on non-urgent procedures in Switzerland in spring 2020, and whether there is evidence that they were in conflict with the recommendations of the SAMW.^3^ To do this, we analyzed the decline in fortnightly adult inpatient hospital admission rates during this period for different types of cardiovascular, orthopedic/musculoskeletal or oncological procedures, and its dependence on patients’ diagnoses and other factors potentially influencing prioritization such as sex, age, insurance status and comorbidity.

## Methods

### Study design and data source

This is a retrospective cohort study based on Swiss routine inpatient data from January 2017 to April 2020. In 2020, Switzerland had a population of 8.67 million.^10^ Data for this study were retrieved from the Medical Statistic of Hospitals (MedStat) 2017 to 2020 of the Swiss Federal Statistical Office.^11,12^ The MedStat collects inpatient data of all Swiss hospitals for the purpose of epidemiological surveillance, healthcare planning, quality control, cantonal comparisons, and others. It contains information on surgical, diagnostic and therapeutic procedures according to the Swiss surgery classification (CHOP codes)^13^ and diagnoses according to the International Classification of Disease, 10th version (ICD-10).^14^ Hospital admissions are categorized as emergencies or elective (or other) entries.

An ethics committee approval was not required for this study, as all data were retrospectively collected and anonymized (Federal Act on Research involving Human Beings, Art. 2).

### Data selection and variable definitions

We extracted the complete set of inpatient admissions from weeks 4 to 49 of the years 2017 to 2019 and from weeks 4 to 17 of the year 2020 (to allow consistent periodization of all years into fortnights), except for ignoring admissions of patients aged <20 years or with unknown insurance status or without a recorded procedure.

In line with the notion that medical urgency of a procedure is generally largely co-determined by the diagnosis, we treated each admission as the combination of the associated main procedure and the patient’s main diagnosis. Where applicable, the extracted admissions were categorized, based on main diagnosis and, secondarily, main procedure as “cardiovascular” (diagnosis code in ICD-10 code range I00–I99: diseases of the circulatory system; procedure code in CHOP chapter 7 [categories 35-39]: operations on the cardiovascular system), “orthopedic/musculoskeletal” (M00–M99: diseases of the musculoskeletal system and connective tissue; 14 [76-84]: operations on the locomotive organs) or “oncological” (C00–D48: neoplasms; no CHOP chapter). Admissions that did not fit into any of these 3 medical specialties as well as those with main procedures belonging to CHOP category 93 (physiotherapy, respiratory therapy, rehabilitation and related procedures) were excluded.

The remaining admissions were stratified according to the admitted patient’s sex×age (4 levels: f[emales aged] 20-64 [years], f ⩾ 65, m[ales] 20-64, m ⩾ 65), insurance status (basic, private [including semi-private]) and comorbidity (Elixhauser score ⩽ 4, ⩾5 [as in Ref.^15^; see below]). Procedure-diagnosis combinations were selected for analysis if there were, in each of the 4 years on average per fortnight, at least 5 corresponding admissions in each sex×age stratum or at least 10 admissions in each insurance status stratum or at least 10 admissions in each comorbidity stratum. For stratified analyses, the respective individual condition was imposed. The selection process is shown in Supplemental Figure S1.

### Data analysis

We used descriptive statistics to summarize all selected inpatient admissions overall and within each group, stratified by COVID-19 year (2020) vs control years (2017-2019) and by calendar weeks 12 to 17 (in 2020, representing the lockdown [= ban] period) vs all other weeks. To describe the severity of all of a patient’s conditions, an Elixhauser comorbidity score^16,17^ was calculated for each admission from the admitted patient’s ICD-10 coded diagnoses using Swiss weights^15^ in the elixhauser_icd10_quan algorithm implemented in the comorbidity(. . .) function from R’s comorbidity package.^18^

We then used multivariable negative binomial regression to quantify the ban-related reductions in inpatient admission rates (ie, the admission rate ratios [ARRs] for ban vs no ban) for specific procedure-diagnosis combinations. The fortnightly rates of hospital admissions were modeled as a function of ban (binary: in place during the lockdown period vs not in place), time (continuous: week number of the fortnight’s first week since the start of the observation period, standardized) and the specific combination (categorical: 15 cardiovascular + 29 orthopedic/musculoskeletal + 9 oncological = 53 levels), with interaction terms to account for combination-specific effects of both ban and time. To further investigate the effects of sex×age, insurance status and comorbidity on the reduction, we stratified the model by adding said variables one at a time to its predictors, along with the necessary interaction terms to allow for combination- and combination×ban-specific effects of the stratifiers.

To quantify and test linear functions of model coefficients (eg, the effect of the ban on the admission rates for procedure-diagnosis combination *c* of patients with insurance status *s*), we used the glht(…) function from R’s multcomp package^19^ and adjusted their 95% confidence intervals for multiple comparisons using confint(. . ., calpha = adjusted_calpha()). To test whether the ban-induced ARRs within one medical specialty (cardiovascular, orthopedic/musculoskeletal, oncological) differed across the levels of a stratification variable (sex×age, insurance status, comorbidity), we used Quade (for the 4-level stratifier sex×age) and Wilcoxon signed-rank tests (for insurance status and comorbidity with their 2 levels each) on the logarithms of the estimated ARRs at the α = 5% level without correcting for multiple testing.

R version 4.3.2 was used for statistical analyses.^20^

## Results

We analyzed a total of 528 261 admissions of 496 456 patients. Admission characteristics and average fortnightly admission rates are shown in Table 1. There were 139 099 cardiovascular admissions (26.3%) for 15 frequent procedure-diagnosis combinations, 326 381 orthopedic/musculoskeletal admissions (61.8%) for 29 frequent combinations and 62 781 oncological admissions (11.9%) for 9 frequent combinations.

**Table 1.** Admission characteristics, by year and period.

Characteristic	COVID-19 year (2020)		Control years (2017-2019)	
	Weeks 12-17: Ban period	Other weeks^a^	Weeks 12-17	Other weeks
*Analyzed admissions (528 261 admissions of 496 456 patients)*				
Number per fortnight, mean (SD)	3546.7 (462.0)	7882.0 (349.5)	7119.4 (645.2)	7033.6 (960.9)
% Admissions of/with				
Female aged ⩽64 years	19.5	22.4	22.7	21.0
Female aged ⩾65 years	26.8	28.3	27.6	28.6
Male aged ⩽64 years	25.9	24.1	25.9	25.4
Male aged ⩾65 years	27.7	25.1	23.9	25.1
Private (vs basic) insurance	28.9	30.8	31.7	31.5
Elixhauser score ⩽4 (vs ⩾5)	59.6	72.6	72.8	72.3
*Analyzed cardiovascular admissions (139 099 admissions of 127 754 patients)*				
Number per fortnight, mean (SD)	1141.3 (95.8)	1901.2 (90.2)	1887.3 (127.7)	1851.4 (202.4)
% Admissions of/with				
Female aged ⩽64 years	7.2	9.3	11.3	10.0
Female aged ⩾65 years	25.4	26.7	26.6	26.7
Male aged ⩽64 years	25.4	22.5	24.1	23.6
Male aged ⩾65 years	42.0	41.5	38.0	39.7
Private (vs basic) insurance	24.9	28.4	28.6	29.1
Elixhauser score ⩽4 (vs ⩾5)	49.2	52.8	57.0	56.1
*Analyzed orthopedic/musculoskeletal admissions (326 381 admissions of 317 064 patients)*				
Number per fortnight, mean (SD)	1746.7 (284.3)	5068.8 (235.2)	4392.2 (463.4)	4355.6 (708.4)
% admissions of/with				
Female aged ⩽64 years	25.3	26.5	26.5	24.5
Female aged ⩾65 years	30.6	30.2	29.6	31.0
Male aged ⩽64 years	29.4	26.3	28.5	27.9
Male aged ⩾65 years	14.7	16.9	15.4	16.7
Private (vs basic) insurance	31.0	31.9	33.0	32.4
Elixhauser score ⩽4 (vs ⩾5)	84.6	89.1	89.6	89.1
*Analyzed oncological admissions (62 781 admissions of 55 198 patients)*				
Number per fortnight, mean (SD)	658.7 (118.5)	912.0 (41.3)	839.9 (83.8)	826.6 (89.2)
% Admissions of/with				
Female aged ⩽64 years	25.7	27.3	28.0	27.2
Female aged ⩾65 years	19.1	21.3	19.4	20.3
Male aged ⩽64 years	17.6	15.4	16.3	16.1
Male aged ⩾65 years	37.7	36.1	36.4	36.4
Private (vs basic) insurance	30.1	29.5	31.9	31.8
Elixhauser score ⩽4 (vs ⩾5)	11.4	22.0	20.6	20.1

Abbreviation: SD, standard deviation.

a Of the COVID-19 year 2020, only calendar weeks 4 to 17 were considered for the analysis.

### Cardiovascular admissions

**Figure 1.**
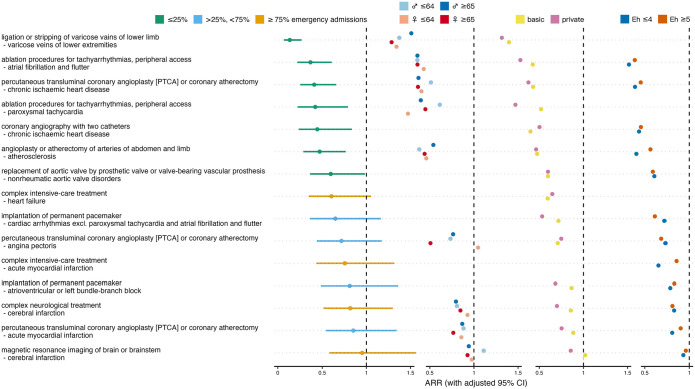
Effect of ban on cardiovascular admissions. Overall admission rate ratios (ARRs) for ban versus no ban with adjusted 95% confidence intervals (CIs), and ARRs stratified for sex×age, insurance status and comorbidity. Colors indicate fractions of emergency admissions per procedure-diagnosis combination, and different strata. Abbreviations: *♀* ⩽ 64, females aged 64 or less; Eh ⩽ 4, patients with Elixhauser comorbidity score ⩽ 4.

Of the 15 cardiovascular procedure-diagnosis combinations analyzed, 7 showed a significant reduction in admission rates during the ban (Figure 1, Supplemental Table S1). The most pronounced decrease was observed for treatment for varicose veins in lower limbs, followed by peripheral access ablation for atrial fibrillation/flutter, and coronary interventions for chronic ischemic heart disease. The smallest decreases were seen in coronary interventions for acute myocardial infarction and in stroke imaging.

In 1 out of the 9 cardiovascular combinations that allowed sex×age-stratified analysis, younger women had the lowest ARRs across all strata, 5 times older women, 2 times younger men, and 1 time older men, which does not indicate a differential effect of the ban on cardiovascular admission rates depending on sex or age. The absence of such a difference was confirmed by the Quade test’s *P*-value of .383. In 10 out of the 14 cardiovascular combinations that allowed analysis stratified by insurance status, privately insured patients had lower ARRs than patients with only basic health insurance, suggesting a significant difference between the 2 insurance status strata, which was confirmed by the Wilcoxon signed-rank test’s *P*-value of .025. In 4 out of the 12 cardiovascular combinations that allowed comorbidity-stratified analysis, patients with more pronounced comorbidity showed lower ARRs than less comorbid patients. Therefore, and as confirmed by the Wilcoxon signed-rank test *P*-value of .110, we found no difference in the ban’s effect on admission rates between the high and low comorbidity strata.

### Orthopedic/musculoskeletal admissions

**Figure 2.**
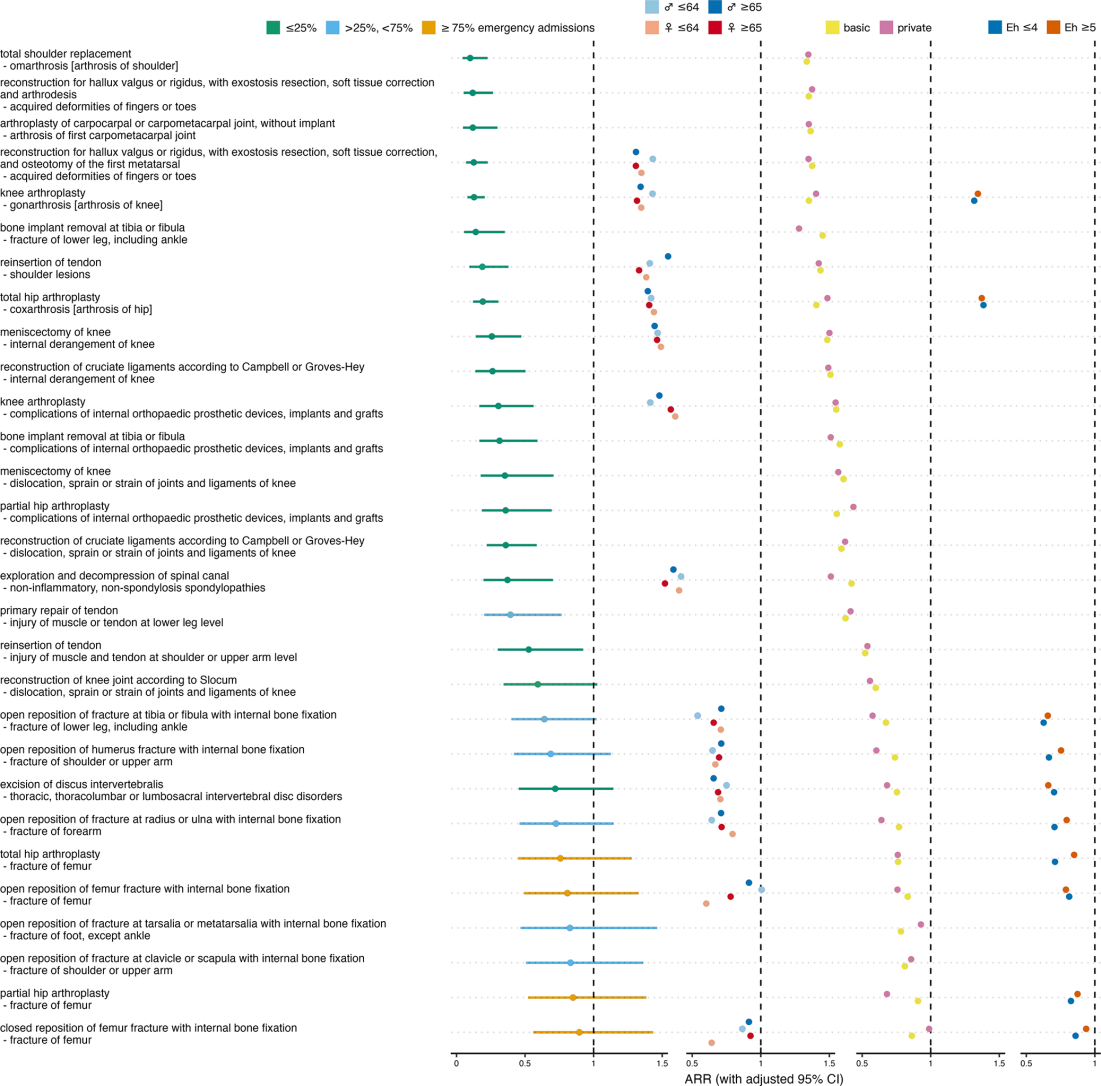
Effect of ban on orthopedic/musculoskeletal admissions. Overall admission rate ratios (ARRs) for ban versus no ban with adjusted 95% confidence intervals (CIs), and ARRs stratified for sex×age, insurance status and comorbidity. Colors indicate fractions of emergency admissions per procedure-diagnosis combination, and different strata. Abbreviations: *♀* ⩽ 64, females aged 64 or less; Eh ⩽ 4, patients with Elixhauser comorbidity score ⩽ 4.

Of the 29 orthopedic/musculoskeletal procedure-diagnosis combinations analyzed, 18 showed a significant decrease during the ban (Figure 2, Supplemental Table S1). The most pronounced decrease was observed for shoulder replacement for omarthrosis, followed by reconstructions for acquired toe deformities, and joint arthroplasty for first carpometacarpal arthrosis. The smallest decreases were seen in partial hip arthroplasty and in closed reposition with internal fixation, both in patients with femur fracture.

In 2 out of 13 orthopedic/musculoskeletal combinations that allowed sex×age-stratified analysis, younger women had the lowest ARRs across all strata, 4 times older women, 4 times younger men, and 3 times older men, which does not indicate a differential effect of the ban on orthopedic/musculoskeletal admission rates depending on sex or age (*P* = .306). In 17 out of 29 orthopedic/musculoskeletal combinations that allowed analysis stratified by insurance status, privately insured patients had lower ARRs than patients with only basic health insurance, which speaks against a difference between the 2 insurance status strata (*P* = .393). In 3 out of 10 orthopedic/musculoskeletal combinations that allowed comorbidity-stratified analysis, patients with more pronounced comorbidity showed lower ARRs than less comorbid patients, showing no difference in the ban’s effect on admission rates between the high and low comorbidity strata (*P* = .084).

### Oncological admissions

**Figure 3.**
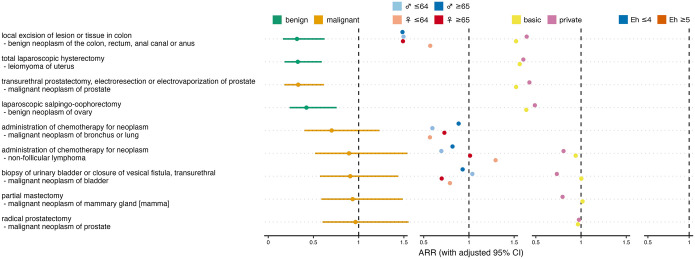
Effect of ban on oncological admissions. Overall admission rate ratios (ARRs) for ban versus no ban with adjusted 95% confidence intervals (CIs), and ARRs stratified for sex×age, insurance status and comorbidity. Colors distinguish between benign and malignant tumors and between different strata. Abbreviations: *♀* ⩽ 64, females aged 64 or less; Eh ⩽ 4, patients with Elixhauser comorbidity score ⩽ 4.

Of the 9 oncological procedure-diagnosis combinations analyzed, 4 showed a significant decrease during the ban (Figure 3, Supplemental Table S1). The most pronounced decrease was observed for local excision of benign colorectal neoplasms, followed by laparoscopic hysterectomy for uterine leiomyoma, and transurethral prostatectomy or electro-resection for prostate cancer. The smallest decreases were seen in partial mastectomy for breast cancer and in radical prostatectomy for prostate cancer.

In 1 out of 4 oncological combinations that allowed sex×age-stratified analysis, younger women had the lowest ARRs across all strata, 1 time older women, 1 time younger men, and 1 time older men, which does not indicate a differential effect of the ban on oncological admission rates depending on sex or age (*P* = .759). In 3 out of 8 oncological combinations that allowed analysis stratified by insurance status, privately insured patients had lower ARRs than patients with only basic health insurance, which speaks against a difference between the 2 insurance status strata (*P* = .641). No oncological procedure-diagnosis combination occurred often enough to allow comorbidity-stratified analysis.

## Discussion

We found no evidence that criteria other than medical urgency affected healthcare resource allocation for hospitalized patients in Switzerland during the COVID-19 pandemic. Except for a stronger decrease of cardiovascular procedures among privately insured patients, we found only minor and non-significant associations with sex×age, insurance status, and comorbidity. Our findings do not refute that the principle of horizontal equity was respected during the ban on non-urgent procedures. However, our study confirmed findings from earlier research which showed that the frequency of inpatient medical procedures was affected to varying degrees by the ban on non-urgent healthcare services during the COVID-19 lockdown.^2,21,22^

In all 3 medical specialties analyzed, we found generally lower decreases in urgent procedures. Among the admissions for cardiovascular reasons, the largest decreases were found with diagnoses that do not necessarily need urgent treatment or can often be treated with medication (varicose veins, atrial fibrillation, chronic ischemic heart disease), while little or no decrease was seen in admission rates for diagnoses requiring urgent procedures (cerebral infarction, acute myocardial infarction). Similarly, we observed significant decreases in less urgent orthopedic procedures in patients with slowly progressing diseases (arthrosis of different joints, acquired deformities of fingers or toes) and at most small decreases of accident-related or critical spinal procedures (fractures of femur, shoulder or arm, intervertebral disk disorders). In oncology, procedure rates for benign tumors decreased substantially, while there were no significant decreases in procedures for malignant tumors, with the notable exception of non-radical therapy of the slowly progressing and thus initially non-urgent prostate carcinoma. Furthermore, the previously reported sharper decrease in elective procedures^2^ compared to those usually performed in emergencies (eg, treatment of osteoarthritis or reconstructive surgery versus treatment of fractures or acute [myocardial or cerebral] infarction) was confirmed by the present analysis, which also suggests that procedures were actually prioritized largely on the basis of medical urgency.

The influence of insurance status on prioritization of medical procedures during the COVID-19 pandemic has only been investigated in healthcare systems (USA,^23^ India^24^) that are hardly comparable to Switzerland. The US, for example, has seen a marked shift in the use of healthcare services based on insurance coverage, with the number of procedures increasing for commercially insured patients and decreasing for Medicare patients,^23^ even though hospitals in the US received extraordinary government subsidies during the COVID-19 pandemic to help them remain financially viable and to mitigate the potential financial motivation to give medically unjustified priority to more lucrative private patients.^25^ In our study, of all tests used to examine associations between decreases in procedure rates and possible influencing factors, only one was significant, namely the one to examine the decrease in cardiovascular procedures according to insurance status. As its *P*-value was not less than 0.025, and considering that we did not correct these 8 tests for multiple testing, this result should be interpreted with caution. A true dependence on the insurance status could suggest that factors other than mere urgency might have played a role in prioritization, for example, hospitals prioritizing certain insurance classes during the pandemic, or conversely, patients in specific insurance classes being over-treated in “normal times,”^26^ resulting in a stronger decrease when only urgent procedures were possible. Procedures with conspicuously pronounced rate differences between different insurance statuses (eg, catheter ablation for paroxysmal tachycardia) may therefore be subject to further investigation.

Further examinations should consider other factors possibly associated with COVID-related reductions in inpatient procedure rates that we could not include in our analysis due to lack of data, but should be discussed as being of interest in their own right or because of their potential to confound the effects of the variables considered in our study. One such factor that has been shown to be associated with healthcare utilization during the COVID-19 pandemic is ethnicity. For example, a study from the US found disparities in the use of telehealth visits by race/ethnicity among veterans with diabetes.^27^ Perhaps even more interesting in the Swiss context is the patient’s socioeconomic status, which is associated with insurance status^28^ but may also independently affect admission rates, for example, if more severely deprived people seek less healthcare in times of crisis. Generally, in most EU and OECD countries, the probability of hospital admission is almost identical across different income quantiles (adjusted for needs),^1^ but there are reports from England that during the first wave of the COVID-19 pandemic, there were socio-economic differences (pro-rich inequities) in the use of general practice consultations and other primary healthcare services, which were effectively narrowed or even eliminated as the pandemic progressed.^29,30^ Contrary, a survey of people aged over 50 years from over 20 European countries found that in most countries, Switzerland included, there were no significant income-related inequalities in postponed or denied medical care.^31^

The raw data in Table 1 show a marked reduction in the fortnightly numbers of (analyzed) admissions of mildly comorbid patients, in line with a previous study of the impact on COVID-19-related restrictions on inpatient care in Switzerland’s most populous canton, Zurich, where the authors observed a shift from lower toward higher case complexity for non-urgent interventions.^32^ A similar reduction in fortnightly admission rates is also noticeable among young women. However, after adjusting for the procedure-diagnosis combination, the decrease was no longer associated with either comorbidity or sex×age. The apparent contradiction is an example of Simpson’s paradox^33,34^: The reduction in raw admission rates is due to non-urgent procedure-diagnosis combinations—such as hysterectomy for uterine leiomyoma—which occur predominantly in women with few comorbidities. Being non-urgent, such combinations show significant rate reductions, and even though the relative reduction is the same across all comorbidity and sex×age strata, in absolute terms it affects more young women with low comorbidity. The decrease in case numbers in the absence of any association with factors other than urgency suggests that during the COVID-19 ban on non-urgent healthcare, hospitals in Switzerland adhered to the principle of horizontal equity by prioritizing procedures according to medical need, despite the high financial pressure from the resulting shift to more complex but, in a DRG-based reimbursement system, less lucrative cases.

### Strengths and limitations

To our knowledge, we were the first to investigate procedure prioritization in inpatients during the first COVID-19 lockdown, taking into account not only the patients’ diagnoses but also considering other potential determinants. Our analysis is based on data from all Swiss hospitals and is thus maximally representative for Switzerland. Noteworthy limitations of our study are: Urgency, being the one justifiable determinant of prioritization and therefore of crucial importance for answering our research question, is nonetheless a vaguely defined concept and was difficult to represent by the variables available. Diagnoses alone, but also our procedure-diagnosis combinations, give an incomplete picture of urgency, and neither comorbidity nor entry mode are satisfactory measures either. This made it difficult to capture the association between urgency and procedure rate decreases with sufficient scientific rigor. From a methodological point of view, it was not very satisfactory that the potential determinants of procedure prioritization could not be analyzed jointly within a single model, as the inclusion of multiple stratifiers would have resulted in sparsely populated strata and too many interactions to estimate. On a similar note, adjustment for factors associated with, for example, insurance status, was not possible due to model complexity and lack of, for example, socio-economic data or data on ethnicity, and we did not examine baseline differences in procedure-diagnosis combinations between different levels of the potential determinants, as it would have been impossible to assess whether any such differences would have been justified. Finally—and obviously—, we could not conclusively prove that there had been no prioritization for unjust reasons at all, as absence of evidence in a limited sample is not evidence to the contrary.

## Conclusion

Our analysis of decreases in adult inpatient admission counts for different types of cardiovascular, orthopedic/musculoskeletal or oncological procedures during the COVID-19 lockdown period, and their dependence on patients’ diagnoses, sex, age, insurance status and comorbidity, did not provide robust evidence that factors other than urgency as inherent in the diagnoses were considered in prioritizing procedures.

## Supplemental Material

sj-docx-1-his-10.1177_11786329241293534 – Supplemental material for Procedure Prioritization During a Nationwide Ban on Non-Urgent Healthcare: A Quasi-Experimental Retrospective Study of Hospital Data in Switzerland Supplemental material, sj-docx-1-his-10.1177_11786329241293534 for Procedure Prioritization During a Nationwide Ban on Non-Urgent Healthcare: A Quasi-Experimental Retrospective Study of Hospital Data in Switzerland by Thomas Grischott, Tarun Mehra, Matthias R Meyer, Oliver Senn and Yael Rachamin in Health Services Insights

sj-xlsx-1-his-10.1177_11786329241293534 – Supplemental material for Procedure Prioritization During a Nationwide Ban on Non-Urgent Healthcare: A Quasi-Experimental Retrospective Study of Hospital Data in Switzerland Supplemental material, sj-xlsx-1-his-10.1177_11786329241293534 for Procedure Prioritization During a Nationwide Ban on Non-Urgent Healthcare: A Quasi-Experimental Retrospective Study of Hospital Data in Switzerland by Thomas Grischott, Tarun Mehra, Matthias R Meyer, Oliver Senn and Yael Rachamin in Health Services Insights

## References

[1] OECD. Health for Everyone? Social Inequalities in Health and Health Systems. OECD Health Policy Studies. 2019. Accessed September 6, 2024. https://www.oecd-ilibrary.org/content/publication/3c8385d0-en

[2] Rachamin Y Meyer MR Rosemann T Grischott T. Impact of the COVID-19 pandemic on elective and emergency inpatient procedure volumes in Switzerland - a retrospective study based on insurance claims data. Int J Health Policy Manag. 2023;12:6932. 36243943 10.34172/ijhpm.2022.6932 PMC10125178

[3] Zentrale Ethikkommission der SAMW. Ausserordentliche Ressourcenknappheit in der stationären Versorgung: Ethische Grundsätze und prozedurale Kriterien für die Verschiebung von Behandlungen. Stellungnahme der Zentralen Ethikkommission (ZEK) der SAMW. Published February 7, 2022. Accessed September 6, 2024. https://www.samw.ch/dam/jcr:9326a1db-ded2-4d98-8dcb-91e6099f0004/stellungnahme_zek_samw_posteriorisierungen_20220207.pdf

[4] Peters O Vuffray C Haslebacher K. Überhang in der stationären Leistungserbringung zu Gunsten der Zusatzversicherten. Published June 10, 2016. Accessed September 6, 2024. https://www.bag.admin.ch/dam/bag/de/dokumente/kuv-aufsicht/stat/articles-et-analyses-aos/stationaere-leistung-zusatzversicherte.pdf

[5] Engelberger K. Akutstationäre Spitaltarife im Zusatzversicherungsbereich - Ein nationaler Tarif- und Kostenvergleich. Published October 2021. Accessed September 6, 2024. https://www.bag.admin.ch/dam/bag/de/dokumente/kuv-aufsicht/stat/articles-et-analyses-aos/stationaere-leistung-zusatzversicherte.pdf

[6] Eidgenössische Finanzmarktaufsicht FINMA. Krankenzusatzversicherer: FINMA sieht umfassenden Handlungsbedarf bei Leistungsabrechnungen. Published December 17, 2020. Accessed September 6, 2024. https://www.finma.ch/de/~/media/finma/dokumente/dokumentencenter/8news/medienmitteilungen/2020/12/20201217-mm-leistungsabrechung-krankenzusatzversicherer.pdf

[7] Mehra T Moos RM Seifert B , et al. Impact of structural and economic factors on hospitalization costs, inpatient mortality, and treatment type of traumatic hip fractures in Switzerland. Arch Osteoporos. 2017;12:7-11. 28013447 10.1007/s11657-016-0302-3

[8] Hochfelder JP Khatib ON Glait SA Slover JD. Femoral neck fractures in New York State. Is the rate of THA increasing, and do race or payer influence decision making? J Orthop Trauma. 2014;28:422-426. 24343251 10.1097/BOT.0000000000000037

[9] Muheim LLS Senn O Früh M , et al. Inappropriate use of arthroscopic meniscal surgery in degenerative knee disease. Acta Orthop. 2017;88:550-555. 28665174 10.1080/17453674.2017.1344915 PMC5560220

[10] Bundesamt für Statistik BFS. Die Bevölkerung der Schweiz im Jahr 2020. Statistik der Schweiz. Published December 23, 2021. Accessed September 6, 2024. https://dam-api.bfs.admin.ch/hub/api/dam/assets/19964430/master

[11] Bundesamt für Statistik BFS. Medizinische Statistik der Krankenhäuser und Statistik der stationären Betriebe des Gesundheitwesens: Detailkonzept. Published October 10, 2014. Accessed September 6, 2024. https://dam-api.bfs.admin.ch/hub/api/dam/assets/230430/master

[12] Bundesamt für Statistik BFS. Medizinische Statistik der Krankenhäuser - Variablen der Medizinischen Statistik. Spezifikationen gültig ab 1.1.2020. Published March 5, 2020. Accessed September 6, 2024. https://dam-api.bfs.admin.ch/hub/api/dam/assets/12167417/master

[13] Bundesamt für Statistik BFS. Schweizerische Operationsklassifikation (CHOP): Systematisches Verzeichnis – Version 2020. Statistik der Schweiz. Published July 31, 2019. Accessed September 6, 2024. https://dam-api.bfs.admin.ch/hub/api/dam/assets/9286150/master

[14] Bundesinstitut für Arzneimittel und Medizinprodukte BfArM. Internationale statistische Klassifikation der Krankheiten und verwandter Gesundheitsprobleme - 10. Revision - German Modification - Version 2018. Accessed September 6, 2024. https://klassifikationen.bfarm.de/icd-10-gm/kode-suche/htmlgm2018/index.htm

[15] Sharma N Schwendimann R Endrich O Ausserhofer D Simon M. Comparing Charlson and Elixhauser comorbidity indices with different weightings to predict in-hospital mortality: an analysis of national inpatient data. BMC Health Serv Res. 2021;21:13. 33407455 10.1186/s12913-020-05999-5 PMC7786470

[16] Elixhauser A Steiner C Harris DR Coffey RM. Comorbidity measures for use with administrative data. Med Care. 1998;36:8-27. 9431328 10.1097/00005650-199801000-00004

[17] van Walraven C Austin PC Jennings A Quan H Forster AJ. A modification of the Elixhauser comorbidity measures into a point system for hospital death using administrative data. Med Care. 2009;47:626-633. 19433995 10.1097/MLR.0b013e31819432e5

[18] Gasparini A. Comorbidity: an R package for computing comorbidity scores. J Open Source Softw. 2018;3:648.

[19] Hothorn T Bretz F Westfall P. Simultaneous inference in general parametric models. Biom J. 2008;50:346-363. 18481363 10.1002/bimj.200810425

[20] R Core Team. R: A Language and Environment for Statistical Computing. R Foundation for Statistical Computing; 2023. https://www.R-project.org

[21] Fahrner R Bähler S Lindner G. COVID-19 lock-down significantly reduced number of surgical presentations in an emergency department. Wien Klin Wochenschr. 2021;133:399-402. 33507348 10.1007/s00508-021-01810-5 PMC7841980

[22] Hübner M Zingg T Martin D Eckert P Demartines N. Surgery for non-Covid-19 patients during the pandemic. PLoS One. 2020;15:e0241331. 10.1371/journal.pone.0241331 PMC7584248 33095834

[23] Wiefels MD Gmunder KN Ruiz JW. Patient income level and health insurance correlate with differences in health care utilization during the COVID-19 pandemic. J Public Health Res. 2023;12:1-8. 10.1177/22799036231160624 PMC9998417 36911537

[24] Krishnamoorthy Y Kuberan D Krishnan M , et al. Impact of health insurance coverage on health care utilization during COVID-19 pandemic: a propensity score matched survey analysis in a target region in India. Int J Health Plann Manage. 2023;38:723-734. 36788661 10.1002/hpm.3620

[25] Wang Y Bai G Anderson G. COVID-19 and hospital financial viability in the US. JAMA Health Forum. 2022;3:e221018. 10.1001/jamahealthforum.2022.1018 PMC9107033 35977260

[26] Struja T Suter F Rohrmann S , et al. Comparison of cardiovascular procedure rates in patients with supplementary vs basic insurance in Switzerland. JAMA Netw Open. 2023;6:e2251965. 10.1001/jamanetworkopen.2022.51965 PMC9860525 36662521

[27] Adhikari S Titus AR Baum A , et al. Disparities in routine healthcare utilization disruptions during COVID-19 pandemic among veterans with type 2 diabetes. BMC Health Serv Res. 2023;23:41. 36647113 10.1186/s12913-023-09057-8 PMC9842402

[28] Altwicker-Hámori S Stucki M. Factors associated with the choice of supplementary hospital insurance in Switzerland - an analysis of the Swiss Health Survey. BMC Health Serv Res. 2023;23:264. 36927575 10.1186/s12913-023-09221-0 PMC10018950

[29] Davillas A Jones AM. Unmet health care need and income-related horizontal equity in use of health care during the COVID-19 pandemic. Health Econ. 2021;30:1711-1716. 33890334 10.1002/hec.4282 PMC8250305

[30] Vestesson EM De Corte KLA Crellin E , et al. Consultation rate and mode by deprivation in English general practice from 2018 to 2022: population-based study. JMIR Public Health Surveill. 2023;9:e44944. 10.2196/44944 PMC10189615 37129943

[31] González-Touya M Stoyanova A Urbanos-Garrido RM. COVID-19 and unmet healthcare needs of older people: did inequity arise in Europe? Int J Environ Res Public Health. 2021;18:9177. 34501767 10.3390/ijerph18179177 PMC8431067

[32] Wirth B Stucki M Thommen C Höglinger M. Auswirkungen der Covid-19-bedingten Einschränkungen auf die stationäre Gesundheitsversorgung im Kanton Zürich. Published July 2022. Accessed September 6, 2024. https://www.zh.ch/content/dam/zhweb/bilder-dokumente/themen/gesundheit/gesundheitsversorgung/spitaeler_kliniken/zhaw_auswirkungen_der_covid_19_bedingten_einschränkungen.pdf

[33] Simpson EH. The interpretation of interaction in contingency tables. J R Stat Soc Series B Stat Methodol. 1951;13:238-241.

[34] Bonovas S Piovani D. Simpson’s paradox in clinical research: a cautionary tale. J Clin Med. 2023;12:1633. 36836181 10.3390/jcm12041633 PMC9960320

