# Supplementary material for: Procedure Prioritization During a Nationwide Ban on Non-Urgent Healthcare: A Quasi-Experimental Retrospective Study of Hospital Data in Switzerland
Source: Health Serv Insights. 2024 Oct 23;17:11786329241293534. doi: 10.1177/11786329241293534 (PMC11503697; doi:10.1177/11786329241293534)
Supplement: sj-docx-1-his-10.1177_11786329241293534 – Supplemental material for Procedure Prioritization During a Nationwide Ban on Non-Urgent Healthcare: A Quasi-Experimental Retrospective Study of Hospital Data in Switzerland [file sj-docx-1-his-10.1177_11786329241293534.docx]

# Supplemental material

Grischott T, Mehra T, Meyer MR, Senn O, Rachamin Y. Procedure prioritization during a nationwide ban on non-urgent healthcare in Switzerland. *Health Services Insights.* Published …

**
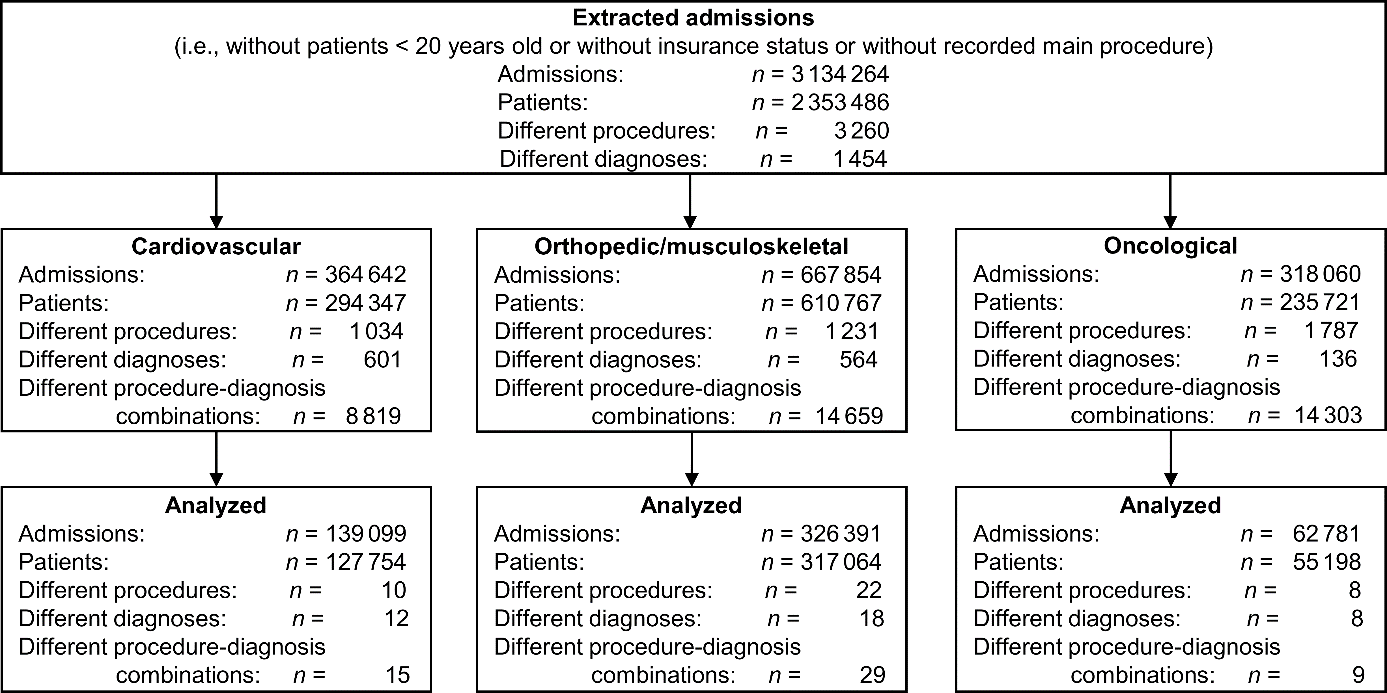
**

**Figure S1**. Data selection flowchart.

This supplementary online content has been provided by the authors to give readers additional information about their work.
